# Malaria Vaccine Adjuvants: Latest Update and Challenges in Preclinical and Clinical Research

**DOI:** 10.1155/2013/282913

**Published:** 2013-04-23

**Authors:** Elena Mata, Aiala Salvador, Manoli Igartua, Rosa María Hernández, José Luis Pedraz

**Affiliations:** ^1^NanoBioCel Group, Laboratory of Pharmaceutics, School of Pharmacy, University of the Basque Country (UPV/EHU), Vitoria-Gasteiz, Spain; ^2^Networking Biomedical Research Center on Bioengineering, Biomaterials and Nanomedicine (CIBER-BBN), Vitoria-Gasteiz, Spain

## Abstract

There is no malaria vaccine currently available, and the most advanced candidate has recently reported a modest 30% efficacy against clinical malaria. Although many efforts have been dedicated to achieve this goal, the research was mainly directed to identify antigenic targets. Nevertheless, the latest progresses on understanding how immune system works and the data recovered from vaccination studies have conferred to the vaccine formulation its deserved relevance. Additionally to the antigen nature, the manner in which it is presented (delivery adjuvants) as well as the immunostimulatory effect of the formulation components (immunostimulants) modulates the immune response elicited. Protective immunity against malaria requires the induction of humoral, antibody-dependent cellular inhibition (ADCI) and effector and memory cell responses. This review summarizes the status of adjuvants that have been or are being employed in the malaria vaccine development, focusing on the pharmaceutical and immunological aspects, as well as on their immunization outcomings at clinical and preclinical stages.

## 1. Introduction

It was almost 50 years ago when the inoculation of attenuated sporozoites evidenced protective immunity and, therefore, the feasibility of developing a cost-effective malaria vaccine. However, the most advanced candidate up to date has only achieved moderate efficacy (30%).

One of the reasons for the slow progress in developing an effective malaria vaccine is the strong capacity of *Plasmodium* parasite to evade host's immune response. This ability derives from the genetic complexity of the pathogen, which exhibits genetic diversity as well as antigenic variation during the multistage life cycle. In consequence, immune responses combining both humoral and cellular responses, that target different asexual stages of the *Plasmodium *live cycle, would be more suitable to achieve host protection against malaria. In addition, vaccines can also be directed to sexual stage for blocking the transmission of the parasite. 

Humoral response induces opsonization of sporozoites, blockage of red cell invasion, and elimination of infected cells directly or through antibody-dependent cellular inhibition (ADCI). On the other hand, cellular response is decisive to produce cytokines and immune mediators (T helper cells (Th)), as well as to kill infected hepatocytes (cytotoxic CD8^+^ T lymphocytes, (CTLs)) [1]. 

Another handicap for malaria vaccine development derives from the traditional strategies of research focused on the antigen-targeting approach, not considering the role of other components of the formulation that can induce and modulate the immune response. Probably several antigen candidates had been abandoned based on the unsatisfactory clinical results obtained because they were inadequately adjuvanted. For example, the most advanced malaria vaccine (RTS,S) did not produce protection when it was formulated with Alum, but it induced moderate protective efficacy of 30% when formulated with adjuvants such AS02 and AS01 [2, 3].

During the last decades, adjuvants have become increasingly important components for the development of vaccines. They are compounds that enhance and direct specific immune responses, classified based on their principal mechanism of action (Table 1). Immunostimulatory adjuvants can increase specific responses by direct stimulation of the immune system, whereas adjuvants that act as delivery systems carry the antigens to the immune cells. The main advantage of the delivery systems is that they allow the coadministration of immunostimulants and more than one antigen into the same system.

This review summarizes the adjuvants evaluated up to date for the development of an efficient malaria vaccine and focuses on their pharmaceutical and immunological properties, as well as their major advances and remarkable results. The paper includes the adjuvants that are already licensed, those approved for clinical use, and those under preclinical research.

To facilitate the followup and comprehension of the paper, a description of malaria antigen candidates is summarized in Table 2, as well as a compilation of malaria vaccine candidates clinically tested and classified as a function of the adjuvant (Table 3).

## 2. Adjuvants for Malaria Vaccines under Clinical Evaluation

This section describes the adjuvants that have been employed during the clinical development of diverse malaria vaccine candidates, considering the most relevant vaccines and clinical trials. 

### 2.1. Alum

Alum, the noncrystalline gel-like forms of aluminum salts, was the first adjuvant approved for human use around 80 years ago [4]. It is a component of numerous licensed vaccines, such diphtheria-tetanus-pertussis (DTP), hepatitis A and B virus (HAV, HBV), human papilloma virus (HPV), *Haemophilus influenza*, and *Streptococcus pneumoniae* [5].

Alum has the capacity to stimulate strong humoral responses (Th2) [6, 7]. The interaction of Alum with the immune system has not been completely clarified and several mechanisms of action have been proposed. First, it was believed that Alum only produced a depot effect and thereby a sustained release of antigen [8, 9]. However, several studies have reported a rapid desorption of this adjuvant from the injection site [10, 11]. What is clear is that the administration of the antigen in a particulate form favors its capture by antigen-presenting cells (APCs) [12, 13]. Alum has also demonstrated its own immunostimulatory capacity [14–16]; a direct or indirect (mediated by dangerous signals such uric acid) ability of Alum to activate NALP3, a component of the inflammasome complex, has been described [7]. This activation leads to caspase-1 activation, proinflammatory cytokine secretion (IL-1*β*, IL-18, IL-33), and monocyte migration to lymph nodes (LNs) for their differentiation into inflammatory dendritic cells (DCs) [17, 18]. However, other studies have reached contradictory results and proposed that the inflammasome activation does not contribute to the adjuvanicity of Alum [19, 20].

Currently, Alum is the most used adjuvant in the clinical evaluation of malaria vaccines. The first malaria vaccine candidate extensively tested in endemic areas was the Alum-adjuvanted SPf66 multistage antigen [21]. With the exception of reducing the incidence of new episodes (28%) [22], results concluded the presence of short-lived antibodies, low cellular responses and no evidence of protection. Therefore, this candidate was abandoned after 10 clinical trials. The preerythrocytic antigen PfCS102 has also been evaluated in combination with Alum, but no success has been achieved [23]. Slightly better results have been obtained with Alum-adjuvanted blood-stage vaccines. MSP1-C1_42_ [24], AMA1-FVO_25-545_ [25], AMA1-C1 [26] and GLURP_85-213_ [27] elicited moderate antibody levels but poor cellular responses (IFN-*γ* secretion, lymphoproliferation, and *in vitro* inhibition of parasite growth) and no protection in field clinical trials. Despite these findings, the clinical research with these antigens continued following their reformulation with other adjuvants and/or combination with other antigens. 

Nowadays other Alum-adjuvanted blood-stage antigens are under clinical trials. It has been shown that EBA-175 RII [28] antigen stimulates functional antibody responses, with certain capacity to inhibit *in vitro* parasite growth and parasite binding to the erythrocytes. MSP3_181-276_ and MSP3_154-249_  also induced considerable antibody-based humoral responses during Phase I trials. In addition MSP3_154-249_ also produced cellular responses including IFN-*γ* secretion and lymphoproliferation, leading to Phase II clinical evaluation [29, 30]. Another Alum-adjuvanted vaccine under Phase II trials is GMZ2 (recombinant protein of MSP3 and GLURP), which elicited considerable antibody levels, including cytophilic ones, cross-reactivity with other malaria antigens, and cell memory during 1 year [31]. The most recent Alum-adjuvanted antigen under clinical evaluation is SE36, which shows promising antibody responses, including 100% seroconversion after the second dose in adult recipients [32].

With regard to sexual stage vaccines, the transmission-blocking Pf25 and Pv25 proteins (expressed on the surface of ookinetes in the mosquito stage of *P. falciparum* and *P. vivax*, resp.) have been clinically evaluated in combination with Alum. They produced poor immunogenicity and Pf25 induced local reactogenicity [33, 34]. A Phase I clinical trial is being conducted with Pf25 conjugated with the recombinant *P. aeruginosa* ExoProtein A (EPA) and adjuvanted with Alum [35], which demonstrated higher specific antibodies and transmission-blocking activity than Pf25/Alum alone [36] in preclinical studies. 

The advantages of Alum are the strong stimulation of the antibody secretion, the extensive experience on clinical safety, the antigen stability, the relative low cost, and the ease to formulate and to scale up. However, it presents significant limitations such as insufficient immunopotentiation in comparison to other adjuvants, incapability to induce appropriate Th1-mediated and CTL cellular responses, and the risk of inducing allergic-type eosinophilic responses and granulomatosis.

### 2.2. Emulsions

Emulsion-based adjuvants include two types of emulsions, water in oil (w/o) and oil in water (o/w). In their majority, they employ squalene, in the oily phase, a natural component of cell membranes and precursor of cholesterol.

#### 2.2.1. MF59

MF59 adjuvant, currently licensed for the influenza vaccine, is a fluid o/w nanoemulsion composed of squalene droplets stabilized with Tween 80 and Span 85. MF59 overcomes Alum adjuvant in eliciting potent and balanced antibody responses. Moreover, it stimulates strong T helper cell responses [37]. Nevertheless, MF59 shows limited ability to induce CD4^+^ Th1 immune responses [38] and polarizes Th2-biased responses [39]. MF59 acts by direct immunostimulation of monocytes, macrophages, and granulocytes to produce cytokines and chemokines [40]. 

Preclinical research on MF59-adjuvanted malaria vaccines did not provide satisfactory results. A study of mice immunization against PvDBP formulated with MF59 demonstrated that it can elicit antibodies as well as IFN-*γ* secretion; however, antigen formulated with Montanide or AS02A achieved enhanced antibody responses with superior titers and higher capacity to block erythrocyte invasion [41]. Moreover, MSP-1_42 _ protein revealed almost no immune response when formulated with MF59 in a mice immunization study [42], low antibodyital and no protection in an Aotus challenge trial [43], as also happened against SERA-1 protein [44]. All these results determined no further progression towards clinical stage with any MF59-based malaria vaccine.

#### 2.2.2. AS03

AS03 adjuvant, registered for human influenza pandemic vaccine, is an o/w emulsion that contains squalene, vitamin E, and Tween 80. It produces strong antibody levels, B cell memory, and CD4^+^ T cell [45] responses but, as other squalene based adjuvants, they are mainly Th2 biased. In addition, vitamin E confers further stimulant properties on AS03 [46] and stimulates sustained antibody responses [47]. The most remarkable use of the AS03 regarding malaria has been its combination with the RTS,S vaccine. Although it elicited a strong antibody response in healthy volunteers, only a moderate protective efficacy of 25% was reached following challenge [48]. 

#### 2.2.3. Montanide

Montanide ISA 51 (ISA-51) and Montanide ISA 720 (ISA-720) are w/o microemulsions composed of squalene stabilized with surfactants [49]. Montanides produce strong antibody secretion, T cell proliferation [50], and balanced Th1/Th2 cytokine profiles [41]. They recruit, activate, and induce migration of APCs to dLNs. Moreover, they interact with cellular membranes favoring the antigen uptake [49]. Furthermore, unlike o/w emulsions, Montanide exerts a depot effect [51]. 

Several ISA-720 adjuvanted malaria vaccine candidates have been or are currently being clinically evaluated. PfCS102 demonstrated very encouraging results at early dose-escalation trials, including the induction of specific and functional antibodies, as well as strong but short-termed CD8^+^ and CD4^+^ immune responses [23], although it did not induce protective immunity after sporozoite challenge [52]. Another Phase I/IIa clinical trial has been conducted with a preerythrocytic antigen, LSA-3, adjuvanted with ISA-720 or Alum. Although no data has been reported yet, LSA-3/ISA-720 has previously demonstrated promising protection on a primate model [53]. 

Regarding blood-stage vaccines, combination B candidate, comprising recombinant proteins from MSP1, MSP2, and RESA, produced strong T cell responses and weak antibody levels in both healthy and field volunteers [54]. However, no significant efficacy on parasite growth rate was shown at challenge trials [55]. Similarly, PfCP2.9 evoked high antibody responses but failed in inducing functional activity against parasite [56]. On the contrary, ISA-720 adjuvanted AMA1-FVO_25-545_ [25], AMA1-C1 [57], GLURP_85-213_ [27], MSP3_181-276_ [29], and MSP2-C1 [58] antigens elicited strong antibody levels, including cytophilic subclasses in some cases. T cell responses were also elicited in healthy volunteers, including *in vitro* peripheral blood mononuclear cell proliferation, IFN-*γ* secretion, and parasite growth inhibition. However, the MSP2-C1/ISA-720 vaccine trial had to be ended prematurely because of the production of local reactogenicity at the injection site. Besides, the development of AMA1-C1 candidate adjuvanted with ISA-720 was stopped owing to stability concerns [57]. Finally, no data are already available from JAIVAC-1 (combination of proteins MSP1_19_ and EBA_175_), the last ISA-720 adjuvanted blood-stage candidate under Phase I clinical trials.

Regarding the clinical use of Montanide on sexual stage vaccines, it is worth mentioning that although Pf25 and Pv25 adjuvanted with ISA-51 elicited moderate to low antibody responses, respectively, the reported severe local reactogenicity and cases of systemic erythema nodosum (higher severe with Pf25) rendered these candidates for further development [59].

The advantage of the Montanides is their capacity to stimulate both humoral and cellular responses. However, they have been associated with critical drawbacks such as pain and unacceptable reactogenicity at the injection site reported in several studies, certain concerns regarding the stability of the antigens, and the need to develop individually extensive and costly specific emulsification procedures for each antigen. In addition, a risk of induction or exacerbation of inflammatory arthritis in genetically susceptible humans has been described for squalene-based emulsions [60]. 

### 2.3. Saponins

Saponins are natural glycosides of steroids or triterpenes widely distributed in plants and animals. Quil-A, an extract of *Quillaja saponaria*, and its derivatives constitute the most extensively used saponins for adjuvant purposes [61]. Although saponins have been widely employed as adjuvants and they are registered for several veterinary vaccines [62], their inclusion into human vaccines has been precluded due to their associated toxicity [63]. QS21, one of the highly purified fractions isolated from Quil-A, exhibits the maximal adjuvanticity with lower toxicity. Saponins stimulate specific humoral and cellular immune responses including Th1 cytokines, cytophilic antibodies, and strong antigen-specific CTL responses [64]. They interact with the cell membrane of APCs gaining the endogenous presentation pathway [65].

The potential of QS21 as an adjuvant for malaria vaccine development was evaluated solely once at clinical stage using the SPf66 multistage antigen. This candidate overcame the immunogenicity elicited when formulated with Alum and it induced higher and longer-lasting antibody levels. However, 2.3% of the individuals developed severe vaccine allergy, an important complication for a prophylactic vaccine [66]. 

Immunostimulating complexes (ISCOMs), whose principal component is Quil-A, comprise micellar cage-like particles of about 40 nm spontaneously formed upon mixing antigens with saponins, phospholipids, and cholesterol [67]. ISCOMs are capable of boosting humoral and cellular responses by both parenteral and mucosal routes [68, 69]. ISCOM adjuvants induce the recruitment and activation of APCs, increase MHC II expression on APCs, enlarge the antigen presentation at dLNs, enhance cross-presentation, trigger the secretion of cytokines such as IL-2, IL-6, and IFN-*γ*, and generate CD4+ and CD8+ CTL responses [67, 70]. 

ISCOM-based malaria vaccines have reported good results during preclinical investigation. An adjuvant trial against the hybrid protein SC_26_42 in rabbits demonstrated that ISCOM formulation elicited high but short-duration antibodies [71]. On the other hand, two fusion proteins including the antigen Pf155/RESA, ZZ-M3 and ZZ-M5, coupled to preformed influenza virus membrane glycoprotein derived ISCOMs induced long-lasting antibody responses comparable to those obtained with Freund's adjuvant [72]. A more recent immunization study against RESA synthetic peptide entrapped into ISCOMs demonstrated the induction of high levels of high-affinity antibodies in mice [73]. Nevertheless, clinical development of ISCOM-based vaccines has been discouraged due to safety concerns, such as the feeling of mild pain at injection site in a Phase I trial [74]. Besides, ISCOMs possess other disadvantages regarding instability, manufacture, and costs.

### 2.4. Virus-Like Particles

Virus-like particles (VLPs) are formed by the self-assembly of recombinant viral capsid proteins, maintaining a similar structure and morphology. VLPs cannot replicate and are noninfectious, as they do not incorporate genetic material, constituting a safer alternative to attenuated viruses. Antigens can be incorporated in several ways, either included into the genetic material that encodes for the capsid proteins or chemically conjugated to preformed VLP [75]. VLPs can elicit strong humoral and cellular responses, which are supported by their capacity to cross-link B-cell receptors [76], as well as to enter endogenous cross-presentation pathway [77]. Nowadays, there are two VLP-based licensed vaccines, one against HBV, expressing HBsAg antigen, and another one against HPV, expressing the major capsid protein L1 [78]. 

Two VLP-based malaria vaccine candidates employing preerythrocytic antigens have been clinically evaluated. ICC-1132 candidate comprises the truncated self-assembling HBV core protein (HBcAg). It includes T1 cell epitopes and B-cell epitopes from immunodominant CS repeat region inserted in the central loop of HBcAg and CSP universal T epitope fused to the C terminus. ICC-1132 formulated with Alum did not elicit optimal antibody responses in magnitude and parasite recognition [79]. Moreover, ICC-1132 formulated with ISA-720 triggered specific antibodies and modest T cell responses, even though no protection was evidenced [80]. 

The other candidate, RTS,S, consists of the RTS hybrid polypeptide from CSP containing B and T cell epitopes fused to the HBsAg (S), assembled as VLPs. Initial trials with RTS,S formulated with Alum, AS03, or AS04 adjuvants did not show encouraging results [48, 81]. Despite this, the strategy to codeliver RTS,S with more clinically advanced adjuvant systems, AS02 and AS01, has led to the most promising malaria vaccine results up to date [2, 3].

The main problems related to the extensive use of VLP-based vaccines are the constraint to incorporate large epitopes owing to increased difficulties for VLP assembling, the biotechnological manufacturing processes instead of chemical synthesis of the antigen, and the requirement of specific particle constructs for each virus-derived VLP and disease. In addition, VLPs have demonstrated a loss of efficiency on boosting doses due to the generation of neutralizing antibodies against capsid proteins [82]. 

### 2.5. Toll-Like Receptors

The pattern recognition receptors (PRRs) present on APCs recognize the pathogen-associated molecular patterns (PAMPs), which are highly conserved molecular structures shared by some microorganisms [83]. PRRs mediate numerous immune mechanisms, such as opsonization, phagocytosis, apoptosis, activation of the complement cascade, and release of cytokines and chemokines [84, 85]. 

The best-known PRR family comprises toll-like receptors (TLRs) [86], which are predominantly expressed by the first, line professional phagocytes (neutrophils, macrophages, and DCs). Ten TLRs have been identified in humans. TLRs 1, 2, 4, 5, and 6 are expressed on cell surface and recognize extracellular microbial structures, whereas TLRs 3, 7, 8, and 9 are expressed intracellularly and recognize viral and bacterial nucleic acids [87]. PAMPs recognition by APCs triggers the transcription of nuclear factor kB (NF*κβ*), interferon regulatory factors (IRF), and activator protein 1 (AP-1), which in turn induces the expression of inflammatory cytokines, type I interferons, and chemokines [88]. Moreover, TLR engagement promotes DCs differentiation and maturation, antigen presentation and upregulation of costimulatory molecules (CD40, CD70, CD80 and CD86) and secretion of cytokines (IL-1, IL-6, IL-12, and TNF) [89, 90]. Depending on the activated TLR, naïve T cells expand and differentiate towards Th1 or Th2 subsets, leading to CD4^+^ or CD8^+^ T cell activation and the induction of high memory T cell responses. In addition, TLR binding on B cells induces their activation, proliferation, and expression of costimulatory molecules [91]. Therefore, TLR ligands play an important role for linking the innate and adaptive responses. 

#### 2.5.1. MPL and Combinations

The 3-O-decylated monophosphoryl lipid A (MPL) immunostimulant is a derivative of the lipopolysaccharide (LPS) endotoxin expressed in the outer membrane of *Salmonella minnesota*. It is a TLR4 agonist and induces a potent stimulation of the Th1 responses, characterized by the secretion of proinflammatory cytokines and cytotoxic antibodies and the activation of CTLs [92]. MPL-including adjuvant formulations (mainly AS04, AS02, and AS01) are currently under clinical evaluation for several vaccines against cancer and other infectious diseases. 

AS04 adjuvant system, which consists of the combination of MPL and Alum, is currently licensed for human HPV and HBV vaccines. In comparison to Alum, AS04 induces earlier and longer-lasting antibodies [93], and it enhances cell-mediated responses [94]. RTS,S candidate has also been clinically tested when formulated with AS04 adjuvant, although low protection was achieved in naïve challenge model [81].

AS02, an o/w emulsion containing MPL and QS21, is currently under clinical investigation for several infectious diseases (malaria, HBV, and *S. pneumoniae*) [95, 96] and cancer [97] vaccines. With regard to malaria, AS02 has been tested with several candidates. The preerythrocytic antigen PfCS102, exhibited higher humoral and cell-mediated responses, including specific CD8^+^ T cell and IFN-*γ* secretion, when AS02 was used instead of ISA-720 [98]. In spite of those data, the adjuvant formulation chosen for further evaluation was the latter one, and it did not result in much success [52]. FMP011 candidate (recombinant LSA-1 from 3D7 strain) induced strong antibody and IL-2/IFN-*γ*-secreting CD4^+^ T cells when adjuvanted with AS02, although no protection was achieved after sporozoite challenge [99]. Remarkably, RTS,S/AS02 candidate showed very high protection (85%) in early trials with naïve volunteers [48]. However, a dramatic decrease in protection was reported in repeated trials (32%) [100]. Field trials in adults revealed high but very short-term protection (71% during first 2 months) [101], but further trials demonstrated only partial protection (32% over 6 months) [102]. On the other hand, trials on children evidenced moderate efficacy against clinical, infection and severe disease (30, 45 and 58%, resp.) during 6 months and a maintained efficacy over 45 months against clinical and severe disease (30 and 39%, resp.) [103]. The reformulated pediatric RTS,S/AS02D has reported 66% of efficacy against infection in infants aged 8–18 weeks after 6 months of followup [104], and an efficacy of 33% against clinical malaria over 14 months of followup [2].

In the case of blood-stage candidates, AMA1-FVO_25-545_ produced greater antibody levels and parasite growth inhibition rate when adjuvanted with AS02 in comparison to Alum or Montanide [25]. FMP1 (recombinant MSP1_42_ from 3D7) formulated with AS02 evidenced no efficacy on a proof-of-concept trial even though it had previously demonstrated safety and immunogenicity [105]. On the contrary, FMP2.1/AS02 (recombinant AMA-1 from 3D7) has shown potent humoral and cellular responses in naïve volunteers and malaria-exposed children [106], and although no protection was achieved in field trials, it induced significant reduction in parasitemia (suggesting partial biological effect) [107], and it is being further evaluated.

AS01 adjuvant system is a liposomal formulation containing MPL and QS21. It is being used in clinical research for the development of malaria, tuberculosis (TB), and HIV vaccines [108, 109]. AS01 elicits potent humoral and cell-mediated responses, including CTL responses. Moreover preclinical data suggest that AS01 surpasses AS02 on its adjuvant effect [110, 111]. Concerning malaria vaccine clinical research, the preerythrocytic candidate FMP011 adjuvanted with AS01 failed to induce protection although immunogenicity was proved, as previously happened with AS02 [99]. On the other hand, RTS,S candidate formulated with AS01 has demonstrated enhanced immunogenicity and protection in comparison to RTS,S/AS02 in naïve adult challenge (50% versus 32%) [100] and in field trials with children 5–17 months of age (53% versus 30%) [112]. A pivotal Phase III has been completed in children/infants (5–17 months and 6–12 weeks, resp.). First data corresponding to the older group showed an efficacy of 55% after a followup of 12 months [113]. However, recent results from the latter group have evidenced only a 30% efficacy [3]. Nevertheless, these are the most promising data during the last decade and they have rendered RTS,S/AS01 as the most advanced malaria vaccine, which is still planned to be reviewed in 2015 for a policy recommendation. With regard to blood stage, FMP2.1 antigen adjuvanted with AS01 evidenced neither protection nor parasitemia-decreasing ability, unlike AS02 [106].

Overall, with the exception of certain local reactogenicity, MPL-based adjuvants are safe and well tolerated. Therefore, their limitations are reduced to the complexity of their composition and the implications related to the inclusion of natural immunostimulants.

#### 2.5.2. Immunostimulatory Oligonucleotides

Synthetic CpG oligodeoxynucleotides (ODNs) are considered as immunostimulant sequences (ISS). They comprise short synthetic DNA molecules containing unmethylated CpG motifs (cytosine phosphoguanosine dinucleotides common in bacteria and virus) [114]. CpG ODNs possess a high potential to induce innate immunity as well as specific humoral and Th1-cell mediated responses [115]. For this reason, CpG ODNs have also been postulated as adjuvants for cancer [116] and allergy [117] vaccines. CpG ODNs mediate their immunostimulatory capacity through the TLR9 (receptors expressed on human plasmacytoid DCs (pDCs) and B cells), promoting Th1-biased CD4^+^ T cell and CTL responses. 

Regarding malaria, CPG7909 has been clinically tested in combination with carrier adjuvants in order to increase and modulate the immune response. AMA1-C1 and MSP1-C1 blood-stage candidates formulated in Alum + CPG7909 induced enhanced specific and functional antibodies that possessed higher capacity for *in vitro* inhibition of parasite growth in comparison to Alum alone [24, 118]. However, the capacity of CPG7909 to induce specific memory B cells was only shown in naïve individuals [119]. Preliminary *in vitro* growth inhibition has also been reported in a more recent trial with BSAM-2 candidate (a mixture of equal amounts of four proteins corresponding to the 3D7 and FVO alleles of MSP1 and AMA-1) formulated into same combination of Alum+CPG7909 adjuvants [120].

Even though clinical trials indicate that CpG ODNs are relatively safe and well tolerated, they have been associated with increased adverse events and local reactogenicity [121]. Moreover, autoimmune responses related to CpG ODNs have been described in preclinical studies [122]. Despite this, it is believed that formulating CpG ODNs in the appropriate adjuvant delivery system may improve their risk-benefit balance and facilitate their approval for human use.

### 2.6. Virosomes

Virosomes are reconstituted membranes formed from enveloped viruses after viral disruption, so they lack the genetic material. The viral envelope from the virosomes is used as a platform to insert other viral or nonviral components by surface adsorption or integration into the lipid membrane [123]. Virosomes have demonstrated to induce both humoral and cellular responses [124]. The currently licensed virosome-based vaccines (HAV and influenza) comprise immunopotentiating reconstituted influenza virosomes (IRIVs). IRIVs are proteoliposomes composed of purified hemagglutinin (HA) and neuraminidase (NA) from influenza virus intercalated within the phospholipid bilayer [125]. 

The presence of the HA in the IRIVs favors the interaction with the immune cells [126], leading to the antigen presentation into MHC class I and II molecules and subsequent activation of both humoral and cellular immune responses [123, 127].

Different IRIV formulations have been evaluated in malaria vaccine clinical trials. Virosomal formulation PEV301 containing AMA49-C1 peptide and PEV302 containing UK-39-peptide have evidenced safety and antibody-based immunogenicity after boosting [128]. Besides, UK-39 immunized individuals developed antibodies able to inhibit the sporozoite migration and hepatocyte invasion *in vitro*. Likewise, coadministration of both formulations, called as PEV3A, did not interfere with the immunogenicity of each formulation [129], and a reduction in the parasite growth rate was reported [130]. Nevertheless, no protection after challenge was observed [131].

### 2.7. Viral Vectors

Viral vectors can act as delivery systems by carrying the genetic material encoding for antigens, which will be expressed after immune cell entering [132]. They induce efficient cellular immunity, Th, and CTL, as a consequence of the expression of encoded antigens through MHC-I pathway, as well as antibody responses [133, 134]. Viral vectors are frequently used in heterologous prime-boost immunization regime, in which different vaccine technologies are alternated as priming and boosting. 

Viral vectors include DNA and RNA viruses, and the most advanced are poxvirus, adenovirus and flavivirus [135]. There is only one viral vector-based vaccine approved for human use (Imojev, a recombinant yellow fever virus-vectored vaccine against Japanese encephalitis). Several virus vectored vaccines are under clinical development (malaria, tuberculosis, HIV, and cancer) [134]. Regarding malaria, viral vectors are becoming one of the most prolific strategies undergoing clinical development. They involve attenuated fowlpox virus strain FP9 and modified virus Ankara (MVA) poxviruses, human/simian adenoviruses (Ad), and their combinations.

#### 2.7.1. Poxviruses

In a Phase Ia trial, FP9/MVA CSP and DNA/MVA CSP candidates did not reach an efficient T cell activation, and no protection was generated after challenge [136, 137]. However, IFN-*γ*-secreting CD4^+^ and CD8^+^ T cells were activated in a posterior Phase Ib trial in Gambia [138], probably due to the natural priming ability of the vectors.

FP9/MVA ME-TRAP and DNA/MVA ME-TRAP strategies induced strong cell-mediated IFN-*γ* secretion in healthy volunteers, biased towards CD4 for F9/MVA regime and CD8 for DNA/MVA [139]. Further studies in endemic areas revealed no protection against F9/MVA ME-TRAP [140], and an efficacy of 10% for reducing the time of infection against DNA/MVA ME-TRAP [141].

FP9/MVA polyprotein (6 antigens) candidate exploits the poxviruses' capacity to encode large inserts. Unfortunately, it failed in challenge exposition although it elicited T cell responses, and it was discontinued [142]. 

#### 2.7.2. Adenoviruses

A clinical trial with human Ad subtype 5 (Ad5) encoding CSP and AMA-1 (two different vectors), called NMRC-M3V-Ad-PfC, could not achieve sterile protection after challenge [143] Moreover, boosting resulted in even less malaria-specific immunogenicity, which could be related to the anti-Ad5 response induced by the priming vaccination. 

Ad35 is less immunogenic than Ad5 but its sero-prevalence in Africa is significantly lower (20% versus 95%). There are currently two vaccines employing Ad35 vector, Ad35.CS and Ad35.CS/RTS,S-AS01, undergoing Phase I/II trials. It has been already described that they produce strong B and T immune responses in primates, including sustained T-cell response for at least 6 months for the latter one [144, 145]. 

Another human Ad with lower seroprevalence (below 20%), Ad26, has entered the clinical stage after showing more potency and long-lasting T cell responses in mice using a heterologous prime-boost regime, Ad35.CS/Ad26.CS [146].

Simian adenovirus ChAd63 has become an alternative to human Ad because there are no significant preexisting neutralizing antibodies in humans. ChAd63 vector in heterologous prime-boost regime using MVA as boosting is currently undergoing several malaria vaccine clinical trials at different stages and targeting preerythrocytic or blood stages (ME-TRAP, MSP1, AMA1, CSP). ChAD63/MVA ME-TRAP (currently under Phase IIb) has demonstrated unprecedented T cell CD4/CD8 effector responses [147] and a degree of protection of 57% after challenge in healthy volunteers [148]. This vaccine strategy has previously shown protection in mice [149], as well as sustained antibodies, T-cell cytokines and improved CD8 multifunctional responses in macaques [150]. ChAD63/MVA MSP1 has demonstrated some antibody secretion and very high mixed CD4/CD8 T cell induction in a Phase I study [151], as well as ChAD63/MVA AMA [152], but no effect on parasite growth rate was observed following challenge [153].

## 3. Adjuvants under Preclinical Development 

The following section describes adjuvants with potential interest for the design of malaria vaccines that have been evaluated in preclinical studies. 

### 3.1. Liposomes

Liposomes are synthetic phospholipid spheres ranging from nano-to micrometer size, comprising uni or multilipid layers, often stabilized with cholesterol [154]. Liposomes are delivery systems that carry antigens or adjuvants encapsulated into the aqueous core (hydrophilic molecules), adsorbed to the surface (lipophilic molecules) or integrated into the lipid layers (amphiphilic molecules) [155]. Although they can provide humoral and cellular immune responses, there is not any liposomal vaccine commercially available. 

Liposomal adjuvanticity is dependent on the number of lipid layers, charge, size, composition, and preparation method. Therefore, its immunogenicity can be modulated by the addition of ligands, antigens, or another type of lipids [123]. The most immunogenic liposomes are the cationic adjuvant formulations (CAF), particularly those made of dimethyl dioctadecyl ammonium (DDA) combined with the modulating and stabilizer glycolipid trehalose dibehenate (TDB). This formulation, known as CAF01, produced cell-mediated responses and promising antibody responses in a mouse model [156]. Immunization studies with *P. yoelii *MSP1_19_ antigen have demonstrated higher antibody levels (IgG1 and IgG2a) *in vitro* cell-mediated IFN-*γ* secretion and earlier parasitemia clearance than Alum. In addition, significant protection was achieved after challenge (lower parasitemia in comparison to unvaccinated group) [157]. In fact, PfAMA-1 and GMZ2 malaria candidates formulated with DDA/TDB almost entered clinical stage, but they were discontinued after toxicological studies [158].

The main drawbacks of liposomes are related to their stability, manufacturing process, and high costs. Besides, they require the inclusion of immunostimulatory molecules. Additionally, pain at injection site can also be a limitation in some liposomal vaccines.

### 3.2. Polymeric Particles

Polymeric particulate delivery systems are spherical structures ranging from nano-to micrometer size and usually made of biodegradable polymers. In addition to the controlled release [159], biodegradable particles can improve the response of poor immunogenic antigens after parenteral and mucosal vaccination [160, 161], and allow the codelivery of immune-stimulating adjuvants [162]. Although there are several microsphere-based marketed products [163], they have been barely studied in clinics and results have not been very encouraging. 

Various polymers have been evaluated as particulate vaccine delivery systems, such as poly-*ε*-caprolactone, poly-*γ*-glutamic acid, starch, alginate, and chitosan. However, poly-lactic-co-glycolic acid (PLGA) [164] is the most advantageous polymer due to its biodegradability and biocompatibility [165], and it has been approved for human use (FDA) for various applications. PLGA particles can elicit strong antibody responses with neutralizing capacity, T cell-mediated responses like lymphoproliferation and cytokine secretion, and CTL responses [166, 167]. 

Several mechanisms of action can support the adjuvant effect of these particles. They can act as a depot at the site of injection, delivering the antigen during long periods of time. On the other hand, they can be taken up by immune cells, a process that can be influenced by the shape, size, hydrophobicity, or charge of the particles. Finally, it has also been proposed that polymeric particles can activate the NALP3 inflammasome for the activation of caspase-1 and secretion of IL1*β*.

With regard to malaria, several preclinical studies have been carried out using polymeric particles entrapping synthetic peptides using different immunization routes. Subcutaneous (sc) route produced humoral and cellular responses comparable to those of IFA adjuvant [168]. Oral administration was comparable to Alum, although the reported cytophilic antibody secretion indicated the capability to induce cellular responses [169]. Nasal immunization evidenced similar antibody secretion compared to CFA/IFA, and it also elicited cytophilic antibody and IFN-*γ* secretion [170]. Intradermal immunization produced a 10-fold increase of the response when compared to the sc route [171], and it also overcame Montanide's subcutaneous administration [167]. 

The major drawback for the progression of polymeric particles towards clinics is the difficulty to scale up and to develop cost-effective individual manufacturing processes under aseptic good manufacturing practices (GMPs).

### 3.3. Polysaccharides

Microbial carbohydrates (glucans, dextrans, glucomannans, galactomannans, levans, and xylans) are signaling molecules for immune system, which can act as potent immunostimulants [172]. 

Regarding malaria, different MSP antigens from the blood stage have been tested in murine models, formulated with MPI (microparticulate *γ*- and *δ*-inulin) and algammulin (hybrid particle resulted from the cocrystallization of Alum and inulin) adjuvants. MPI induced an immune response comparable to CFA in terms of antibody levels (total and subclasses), *in vitro* splenocyte activation (secretion of IFN-*γ* and IL-12), and protection against challenge in mice [173]. In addition to the safety and immunological properties, MPI possesses other considerable advantages, such as heat stability, long shelf life, and extremely endotoxin-free purity.

### 3.4. TLR Immunostimulant Ligands

As it has been mentioned before, TLR agonists exhibit high potential for prophylactic and therapeutic vaccine purposes for inflammatory diseases and cancer [174]. In addition to MPL and CpG, other TLR ligands and synthetic analogues are currently under development.

#### 3.4.1. Imidazoquinolines

Imidazoquinoline compounds comprise the small synthetic molecules imiquimod (R-837) and resiquimod (R-848), which are TLR7 and TLR7/8 ligands, respectively [175]. It has been shown that imidazoquinolines can improve both antibody and T cell responses following diverse administration routes [176, 177]. In addition, topical imiquimod has demonstrated efficacy in human leishmaniasis [178], and it is already licensed for the treatment of malignant and nonmalignant skin disorders. With regard to malaria, the topical administration of imiquimod with the PfCS peptide elicited strong parasite-specific antibody production, CD4^+^ T cell responses, and protection in a rodent challenge model [179].

#### 3.4.2. Flagellin

Flagellin, the main component of flagellar structure in motile bacteria, is recognized by TLR5 and acts as a potent immune activator [180]. 

Flagellin has been evaluated in combination with several malaria recombinant vaccine candidates. Immunization with *P. vivax* MSP-1 (PvMSP1_19_) fused to flagellin elicited strong, functional, and long-lasting antibody-mediated responses, similarly to PvMSP1_19_ emulsified with CFA [181]. However, the immune response was biased towards Th2. This fact could be modulated by the inclusion of additional adjuvants such as TLR9 agonists. Besides, immunization with *P. falciparum* PfMSP1_19_ fused to flagellin induced high antibody levels that efficiently inhibited the *in vitro *parasite growth [182]. Moreover, the CS_280-288_ protein (CD8^+^ T cell epitope from the CSP) from *P. yoelii* induced CS-specific CD8^+^ T cell responses when combined with flagellin and in the absence of any conventional adjuvant [183]. 

#### 3.4.3. TLR3 Agonists

TLR3 is activated by the double-stranded RNA (dsRNA) produced during the replication of most viruses, and their agonists can be considered as potential adjuvants for vaccines targeting strong cellular immune responses. Polyinosinic:polycytidylic acid (polyI:C) is a synthetic analogue of viral dsRNA molecules that activates TLR3 and other non-TLR PRRs [184]. 

With regard to malaria, PfCSP *plus* polyI:C produced specific robust and functional antibodies, as well as improved CD4^+^ T cell responses in comparison to RTS,S/AS01B vaccine in primates [185]. These data support the fact that an appropriate formulation can enhance CD8^+^ T cell priming (which plays a major role on liver stage), providing an alternative for the development of a preerythrocytic vaccine. The major drawback of polyI:C could be its toxicity, as it has been reported in clinical studies of leukemia [186]. For this reason, developing polyI:C derivatives with improved activity and safety, such as PolyICLC or PolyI:C_12_U, has become a priority in this field.

#### 3.4.4. Synthetic TLR4 Ligands

TLR4 receptors are present in DCs, macrophages, and other nonimmune cells. The TLR4 agonist glucopyranosyl lipid A (GLA) is a new synthetic lipid A. GLA can be formulated in solution or as an o/w stable emulsion (GLA-SE). It has been recently reported that GLA-SE produces high immune responses in terms of total IgG and IgG2 antibodies levels, parasite-recognizing antibodies, IFN-*γ* secretion and number of long-lived plasma cells in comparison to other TLR agonists in a murine immunization study with GMZ2 malaria candidate [187]. 

 On the other hand, OM-174, a chemically synthesized soluble triacylated partial structure of lipid A, has demonstrated to elicit strong antibody and CTL responses in mice when combined with the *P. berghei *CSP long-synthetic-peptide (LSP) based malaria vaccine [188]. 

#### 3.4.5. IC31

IC31 is a novel TLR9 agonist that combines the immunostimulatory effect of a cationic antimicrobial peptide (KLKL5KLK) and a synthetic ODN containing deoxy-Inosine/deoxy-Cytosine (ODN1a) but not CpG motifs [189]. Apart from the TRL9 activation, IC31 can provide prolonged antigen exposition periods through the complexation of its two components, forming a depot [190]. A strategic collaboration has been established between Intercell (IC31's proprietary) and PATH Malaria Initiative to evaluate the immunological responses to malaria recombinant antigens in combination with IC31 adjuvant.

### 3.5. Other Adjuvants in Development

#### 3.5.1. CoVaccine HT

Synthetic carbohydrates comprising polysaccharides *plus* lipidic and sulphate groups have demonstrated an interesting immunopotentiation capacity [191]. CoVaccine HT is an o/w emulsion-based vaccine adjuvant consisting of synthetic sucrose fatty acid sulphate esters immobilised inside the oily droplets of the submicron squalane in water emulsion [192]. In accordance with other adjuvants mimicking LPS, the adjuvanticity of CoVaccine HT is mediated through TLR4 signaling, but other mechanisms are also involved since it interacts with DCs independently of binding to TLR4 [193]. 

Different AMA-1 candidates formulated in CoVaccine HT have been tested in macaques. These candidates improved the antibody secretion and their functionality comparing to Montanide ISA 51 [194], and they also elicited protection after challenge [195]. CoVaccine HT is currently under clinical trials, given alone in healthy volunteers for dose escalation studies and in patients for the development of two therapeutic vaccines (angiotensin therapeutic vaccine and prostate cancer). Importantly, CoVaccine HT's manufacturing is a scalable process that can be done following GMPs. 

#### 3.5.2. 7DW8-5 Galcer

Alpha-galactosylceramide (*α*-GalCer), a glycolipid composed of *α*-linked sugar and lipid moieties, is a well-known ligand that binds CD1d, an MHC class I molecule expressed in most monocytes, macrophages, DCs, and B cells and that it is recognized by invariant NK T (iNKT) cells [196]. Upon recognition, *α*-GalCer activates iNKT cells to produce Th1 (IFN-*γ*) and Th2 cytokines (IL-4), which, in turn, activates immune cells including DCs, NKs, B cells, and CD4^+^ and CD8^+^ T cells [197]. *α*-GalCer has evidenced raised immunogenicity and efficacy in preclinical vaccines against several infections [196], including malaria, for which enhanced specific CD8^+^ T cell responses and protective immunity were reported [198].

Several analogues of *α*-GalCer with different Th1/Th2 patterns have been developed. Nowadays, 7DW8-5 has emerged as the most important *α*-GalCer derivate compound, which has demonstrated the strongest adjuvanticity (notably increased for T cell responses but less pronounced for humoral ones) and protective immunity in adenovirus-based HIV and malaria vaccines in murine models [197, 199]. Although glycolipid compounds have progressed to clinical phase as immunotherapeutics for the treatment of cancer and hepatitis B and C, they are not licensed as vaccine adjuvants yet. Nevertheless, they have some benefits such as they allow vaccine dose-sparing and a relative nonexpensive manufacturing process [199].

#### 3.5.3. AFCO1 and AFPL1

AFPL1 is a detergent-extracted outer membrane vesicle (proteoliposome (PL)) of *N. meningitidis B*, and AFCO1 is a particulate derivative presenting multilayer tubular structure. Both AFPL1 and AFCO1 act as delivery systems and have inherent adjuvant capacities (immunopotentiation and immunomodulation). In fact, they contain meningococcal protective antigens (LPS and porins) and allow the packaging of other antigens and PAMPs [200]. Although the antigen can be incorporated during the manufacturing process, their simple coadministration with antigens has also been successfully performed. 

AFCO1 has been used in malaria immunization studies with MSP antigens. It induced strong antibody and T cell responses, comparable to CFA, and stimulated the release of specific cytophilic antibodies, IFN-*γ* and CD4^+^ and CD8^+^ T cell proliferation [201], which is in accordance with previously reported Th1 polarization. 

## 4. Perspectives and Final Conclusions

Currently there is a greater awareness of using suitable adjuvants to develop new effective vaccines. However, there is still a tendency to employ the few approved ones with the wrong intention to rapidly progress to clinical stage. In the case of malaria, a protective vaccine requires antigen-specific B and T helper cell responses, CTL responses, and long-lasting B and T memory cell production. Conventional adjuvants such as Alum cannot induce the immune response needed, and there is not a scientific basis for undergoing clinical trials with those adjuvants. Several reasons can lead to the widespread use of traditional adjuvants; sometimes researchers cannot properly formulate their discovered antigens, or they find difficulties to access to the novel adjuvants. On the other hand, regulatory authorities such as FDA do not approve adjuvants as a product alone, but as a part of a vaccine formulation comprising a determined combination of antigen(s) *plus* adjuvant(s). Each antigen/adjuvant combination requires a complete product development, which restrains the progression of those adjuvants for new vaccine applications [78]. Fortunately, several initiatives have been created during the last years with the aim to promote, rationalize, unify and share the efforts dedicated to the research on new adjuvants [202].

It is essential to know how adjuvants work in order to determine their role in vaccine formulations and to design successful vaccines. The recent advances for clarifying the immune pathways involved in the modulation of the host protective immune response have led to a better understanding of the immunological mechanisms of adjuvants. For instance, new insights on Alum adjuvanticity have been described more than 80 years after its approval. Furthermore, these steps forward have also promoted the discovery of new improved adjuvants. 

One of the greatest progresses corresponds to the discovery of TLRs and other innate receptors with the capacity to link innate and adaptive immunity, resulting in the development of a new generation of adjuvants. Besides, the better knowledge of the molecular structure of TLRs is allowing their substitution by synthetic analogues, overcoming some of the concerns related to their potency, toxicity, and manufacturing problems. In addition, other non-TLR PAMPs with potential as vaccine adjuvants have also been discovered, such as RIG-I-like receptors (RLRs), NOD-like receptors (NLRs), and C-type lectin receptors (CLRs). 

On the other hand, it is being assumed that a single adjuvant is not enough to elicit protective immunity against certain infectious diseases, such as malaria, which require pluripotent immune responses, adapted to the pathogen and to the targeted population. The combination of multiple adjuvants with different mechanisms of action has been proposed to modulate the interaction between the innate and the adaptive responses. Moreover, the effectiveness of the adjuvant combinations should be based on a synergy between immune response enhancers and delivery systems. The most successful novel combination up to date corresponds to AS formulations. In fact, AS04 enhances humoral and cellular responses [94]; AS02 has yielded 32% of protection in malaria field clinical trials using the RTS,S antigen [102]; AS01 has improved AS02's response demonstrating stronger humoral and cell-mediated responses, and enhanced protection against malaria [100]. Other TLR combinations have also exhibited high immunogenicity. For example, the combination of TLR3/4 with TLR7/8/9 increases Th1 CD4^+^ T cell priming [203]; the mixture of TLR3* plus* TLR9 develops specific CD8^+^ T cell responses [204]; and TLR5 combined with TLR9 elicits more balanced Th1/Th2 responses against extracellular pathogens [205]. 

As it has been mentioned before, TLRs can be administered included into a particulate vaccine delivery system. For example, microparticles containing TLR4 ligands produce superior immune responses than immunopotentiators administered in solution [206], and microparticles coated with TLR3 agonist (polyI:C) induce DCs maturation [207].

Despite the difficulties regarding both immunological and socioeconomic aspects that malaria eradication arouses, promising results have been achieved during last decade, like a diminution of the mortality by 25% between 2000 and 2010 [208]. A malaria vaccine could represent a key cost-effective intervention for this purpose. RTS,S/AS01, the most advanced candidate, achieves protective efficacy in children ranging 30–50% [2, 3] and it is believed that it will represent the first-generation malaria vaccine (WHO recommendation expected by 2015). Nevertheless, it would be still necessary to develop a second-generation vaccine showing improved efficacy (at least 75%). Thus, malaria vaccines' efficacy could be successfully improved by new combinations of the existing adjuvants with the novel ones, developed based on emerging immunological targets.

## Figures and Tables

**Table 1:** Classification of adjuvants by their principal mechanism of action.

Immunostimulants	Delivery systems
Saponins Polysaccharides TLR ligands Cytokines	Alum Emulsions Liposomes ISCOMs Polymeric particles Virosomes VLPs Viral vectors

**Table 2:** Description of malaria antigen candidates.

Life cycle-stage	Vaccine	Description
Preerythrocytic	CSP	Circumsporozoite protein exhibited at the sporozoite surface
	PfCS102	282–383 sequence of the C-terminal region of CSP from *P. falciparum* NF54 strain
	ICC-1132	Universal T and repetitive B/T epitopes from CSP fused to HBcAg and autoassembled as VLPs
	RTS,S	CSP C-terminal extreme containing B and T cell epitopes fused to HBsAg and assembled as VLP
	PEV302	Virosome containing UK-39 peptide corresponding to the immunodominant NANP repeat region of CSP
	LSA-3	Liver stage antigen 3
	FMP011	Recombinant protein of LSA-1 from 3D7 strain
	ME-TRAP	Multiepitope (ME) consisted of preerythrocytic fusion antigen consisting of 17 B cell, CD4+, and CD8+ T cell epitopes from six *P. falciparum* antigens fused to the T9/96 allele of (thrombospondin-related adhesion protein) preerythrocytic antigen (TRAP)

Blood stage	MSP1-C1_42_	Combination of the alleles FVO and 3D7 of the 42Kda fragment of the merozoite surface protein 1 (MSP-1)
	AMA1-FVO_25–545_	Recombinant 25–545 sequence of the merozoite apical membrane antigen 1 (AMA-1) from FVO strain
	AMA1-C1	Combination of equal mixtures of the recombinant AMA-1 from FVO and 3D7 strains
	EBA_175_ RII	Region II domain of the erythrocyte-binding antigen 175 parasite protein
	MSP3_181–276_	C-terminal conserved region from MSP- 3 from FC27 strain
	MSP3_154–249_	C-terminal conserved region from MSP-3 from 3D7 strain
	SE36	Recombinant molecule of serine repeat antigen 5 (SERA5)
	PvDBP	*P. vivax* Duffy binding protein, which binds the Duffy blood group antigen as the obligate receptor for erythrocyte invasion
	Combination B	Combination of recombinant proteins from MSP1, MSP2, and RESA (ring-infected erythrocyte surface antigen)
	PfCP2.9	Recombinant protein consisted of domain III of AMA1 and MSP1_19_ from 3D7 and FVO strains, respectively
	MSP2-C1	Combination of recombinant allelic MSP-2 from 3D7 and FC27
	JAIVAC-1	Combination of proteins MSP1_19_ and EBA_175_
	SC_26_42	Hybrid antigen containing the C-terminal fragment of *P. falciparum* precursor to the major surface antigens (PMMSA) and the tetrapeptide repeats of CSP
	FMP1	Recombinant MSP1_42_ from 3D7 strain
	FMP2.1	Recombinant AMA-1 from 3D7 strain
	BSAM-2	A mixture in equal amounts of four proteins corresponding to the 3D7 and FVO alleles of MSP-1 and AMA-1
	PEV301	Virosome containing AMA_49_-C1 peptide derived from loop I of domain III of AMA-1

Sexual stage	Pf25	Protein expressed on the surface of ookinetes of *P. falciparum *
	Pv25	Protein expressed on the surface of ookinetes of *P. vivax *

Multistage	SPf66	Three blood-stage sequences and repetitive sequences of preerythrocytic CSP
	GLURP_85–231_	85–231 sequence of glutamate rich protein (GLURP) expressed both in preerythrocytic and blood stage
	GMZ2	Recombinant protein of MSP3 and GLURP
	PEV3A	Combination by coadministration of PEV301 and PEV302
	Polyprotein	Long polyprotein consisted of 6 antigens; LSA-3, sporozoite threonine and asparagine-rich protein (STARP), liver stage exported protein 1 (Exp-1), preerythrocytic/sexual stage Pfs16, TRAP, and LSA-1
	NMRC-M3V-Ad-PfC	Combination of two human adenoviruses Ad5 encoding CSP and AMA-1, respectively

**Table 3:** Malaria vaccine candidates clinically tested classified as a function of the adjuvant.

Adjuvant	Antigen	Life cycle stage	Clinical stage
Alum	SPf66	Multistage	Short-term antibodies, low cellular response. Nonreproducible reduction of incidence (28%) [22]. Candidate discontinued, antigen reformulated
	PfCS102	Preerythrocytic	Low and nonfunctional antibodies [23]. Candidate discontinued, antigen reformulated
	ICC-1132	Preerythrocytic	Low and non-functional antibodies [79]. Candidate discontinued, antigen reformulated
	RTS,S	Preerythrocytic	No efficacy on challenge trials [81]. Candidate discontinued, antigen reformulated
	MSP1-C1_42_	Blood stage	Moderate antibody levels, poor cellular response. No efficacy field trials [24]. Candidate discontinued, antigen reformulated
	AMA1-FVO_25–545_	Blood stage	Moderate antibody levels, poor cellular response. No efficacy field trials [25]. Discontinued candidate, antigen reformulated
	AMA1-C1	Blood stage	Moderate antibody levels, poor cellular response. No efficacy field trials [26]. Candidate discontinued, antigen reformulated
	GLURP_85–231_	Multistage	Moderate antibody levels, poor cellular response. No efficacy field trials [27]. Candidate discontinued, antigen reformulated
	EBA-175 RII	Blood stage	Functional antibodies, parasite growth inhibition, and parasite binding [28]
	MSP3_181–276_	Blood stage	Humoral response [29]
	MSP3_154–249_	Blood stage	Humoral response, parasite recognition. IFN-*γ* and lymphoproliferation [30]. Progressed to Phase II
	GMZ2	Multistage	Cytophilic antibodies, cross-reactivity, cell memory for 1 year [31]. Progressed to Phase II
	SE36	Blood stage	Antibody response [32]
	Pf25	Sexual stage	Poor immunogenicity, reactogenicity [59]. Reformulated by conjugation to *P. Aeruginosa* EPA recombinant protein [35]
	Pv25	Sexual stage	Poor immunogenicity [59]

AS04	RTS,S	Preerythrocytic	No protection in challenge trials [81]

AS03	RTS,S	Preerythrocytic	Strong antibody level. Moderate efficacy (25%) in challenge trials [48]

QS21	SPf66	Multistage	Higher and longer-lasting antibody levels than Alum. Allergy [66]. Antigen discontinued

Montanide ISA-720	PfCS102	Preerythrocytic	Specific and functional antibodies, short-term cellular response [23]. No efficacy in challenge trials [52]
	ICC-1132	Preerythrocytic	Strong antibody response, low T cell response. No efficacy in challenge trials [80]
	Combination B	Blood stage	Low antibody response, strong T cell response in field trials. No parasite inhibition in challenge trial [54, 55]
	PfCP2.9	Blood stage	High but no functional antibodies [56]
	AMA1-FVO_25–545_	Blood stage	Strong antibody response, PBMC proliferation, IFN-*γ*, parasite inhibition in malaria-naïve adults [25]
	AMA1-C1	Blood stage	Strong antibody response, PBMC proliferation, IFN-*γ*, parasite inhibition in malaria-naïve adults. Antigen instability reported [57].
	GLURP_85–231_	Multistage	Strong antibody response, PBMC proliferation, IFN-*γ*, parasite inhibition in malaria-naïve adults [27]
	MSP3_181–276_	Blood stage	Strong antibody response, PBMC proliferation, IFN-*γ*, parasite inhibition in malaria-naïve adults [29]
	MSP2-C1	Blood stage	Strong antibody response, PBMC proliferation, IFN-*γ*, parasite inhibition in malaria-naïve adults. Unacceptable reactogenicity [58]
	Pf25	Sexual stage	Moderate antibody levels. Severe reactogenicity and erythema nodosum [59]
	Pv25	Sexual stage	Low antibody levels. Reactogenicity [59]

AS02	PfCS102	Preerythrocytic	Higher humoral and cell-mediated response than ISA-720 [98]
	FMP011	Preerythrocytic	Strong antibody and CD4+ T cell response. No efficacy in challenge trials [99]
	RTS,S	Preerythrocytic	Partial protection in challenge and in adult field trials (32%) [100]. Partial protection against clinical and severe malaria (30 and 39%) in children after 45 month followup [103]. Partial protection against clinical malaria (33%) in infants after 14-month followup [2]
	AMA1-FVO_25–545_	Blood stage	Higher antibody levels and parasite inhibition than ISA-720 [25]
	FMP1	Blood stage	Immunogenicity. No efficacy in challenge trials [105]
	FMP2.1	Blood stage	Strong humoral and cellular responses, naïve adults and field children. Reduction of parasitemia. No protection in field trials [106, 107]

AS01	FMP011	Preerythrocytic	Immunogenicity. No efficacy in challenge trials [99]
	RTS,S	Preerythrocytic	Moderate protection in naïve adults (50%) [100]. Efficacy of 55% in children 5–17 months and 30% in infants 4–16 weeks after a followup of 1 year [3, 113]
	FMP2.1	Blood stage	No protection, no parasitemia reduction [106]

Alum + CPG7909	AMA1-C1	Blood stage	Higher specific and functional antibodies and parasite inhibition than Alum alone [103]. Memory B cell only in nonexposed individuals [119]
	MSP1-C1_42_	Blood stage	Higher specific and functional antibodies and parasite inhibition than Alum alone [24]. Memory B cell only in nonexposed individuals [119]
	BASM-2	Blood stage	Preliminary stage, parasite growth inhibition [120]

Virosomes	PEV302	Preerythrocytic	Safety, immunogenicity after boosting [128]
	PEV301	Blood stage	Safety, immunogenicity after boosting. Inhibition of sporozoite migration and hepatocyte invasion [127]
	PEV3A	Multistage	Immunogenicity related to PEV301 and PEV302, additionally to parasite growth inhibition [129, 130]. No protection in challenge model [131]

Viral vectors	F9/MVA CSP	Preerythrocytic	No efficient induction of effector T cells in naïve volunteers but moderate in endemic population. No protection [136, 138]
	DNA/MVA CSP	Preerythrocytic	No efficient induction of effector T cells. No protection [137]
	FP9/MVA ME-TRAP	Preerythrocytic	Strong CD4+ IFN- *γ* secretion in healthy volunteers [139]. No protection in field trials [140]
	DNA/MVA ME-TRAP	Preerythrocytic	Strong CD8+ IFN-*γ* secretion in healthy volunteers [139]. Low protection against infection (10%) in field trials [141]
	F9/MVA polyprotein	Multistage	T-cell responses. No protection after challenge [142]
	NMRC-M3V-Ad-PfC	Multistage	Safety, specific immunogenicity prevented after boosting. No protection after challenge [143]
	Ad35.CS/RTS,S-AS01	Preerythrocytic	Not published human protection data
	Ad35.CS/Ad26.CS	Preerythrocytic	Not published human protection data
	ChAD63/MVA ME-TRAP	Preerythrocytic	CD4/CD8 mixed effector response [147]. Certain protection level in 57% of individuals after challenge [148]
	ChAD63/MVA MSP1	Blood stage	CD4/CD8 mixed effector response, no effect on parasite growth after challenge [151, 153]
	ChAD63/MVA AMA1	Blood stage	CD4/CD8 mixed effector response, no effect on parasite growth after challenge [152, 153]

VLPs adjuvants, ICC-1132, and RTS,S are considered within delivery adjuvants with which they were coadministered (Alum, Montanide ISA-720, adjuvant systems (AS)).
